# Comparing the Psychometric Properties of Two Japanese-Translated Scales of the Free Will and Determinism-Plus Scale

**DOI:** 10.3389/fpsyg.2021.720601

**Published:** 2021-10-05

**Authors:** Takayuki Goto

**Affiliations:** School of Human Cultures, The University of Shiga Prefecture, Hikone, Japan

**Keywords:** free will, determinism, translation of the psychological scales, psychometric properties, FAD-plus

## Abstract

The free will and determinism-plus scale (FAD-Plus) is one of the most widely used scales to assess the lay belief of people in the existence of free will and deterministic world views. Past research has translated FAD-Plus into various languages for non-English speaking populations, and there exist two Japanese translations of FAD-Plus: the FAD+ and the FAD-J. This study aimed to compare the psychometric properties of FAD+ and FAD-J. Results revealed that while both FAD+ and FAD-J consist of the same four subscales as the original FAD-Plus, some differences exist in the item-level psychometric characteristics. In general, as for the construct validity, although results supported that both scales can assess almost the same construct in terms of the functionalities, FAD-J tends to be slightly more likely to replicate the correlations obtained in the previous research.

## Introduction

Most people believe in the concept of free will; that is, people think that they have the capacity to choose and control their actions (Nahmias et al., 2005; Carey and Paulhus, 2013). Belief in free will is thought to be essential for people to be responsible for their choices and actions (Nahmias, 2012). Believing in one's own free will allows one to deliberate, regulate, and reflect on his/her choices and actions. In addition, believing in the free will of others allows one to feel that others should be held responsible for their choices and actions. Recently, psychologists have revealed that the lay belief in free will is related to psychological processes underlying self-regulation and attitude toward criminal punishment [see Feldman (2017) for a review].

The development of scales to assess the belief in free will is essential to understanding their psychological functions. Previous researchers have developed some scales assessing belief in free will, including the free will and determinism-plus scale (FAD-Plus; Paulhus and Carey, 2011), the free will and determinism scale (Rakos et al., 2008), and the free will inventory (Nadelhoffer et al., 2014). Among these scales, the FAD-Plus is known to be the most widely used scale among researchers (see Liu et al., 2020). There exist two Japanese-translated versions of the FAD-Plus, independently developed by different researchers (Watanabe et al., 2014; Goto et al., 2015), namely the FAD+ and the FAD-J. Although both of these scales have been independently developed and validated, due to the differences in the creation process and the number of items, it is not clear whether they can assess the same concept. The aim of the present study is to investigate whether these two Japanese-translated scales assess the same construct (that is, belief in free will and determinism) by directly comparing the psychometric properties of these scales.

### The Psychological Function of Free Will Beliefs

There are two main approaches to examining the functions of free will beliefs. One is an experimental approach in which researchers weaken the belief of participants by exposing them to texts with anti-free will (i.e., deterministic) world views. In Vohs and Schooler (2008), the first study to develop this experimental approach, those who read anti-free will texts were more likely to cheat on a test compared with those who did not. Subsequent researchers have used such anti-free will manipulations, reporting that weakening free will beliefs increased aggressive behaviors (Baumeister et al., 2009), conformity (Alquist et al., 2013), and prejudice (Zhao et al., 2014). These consequences of weakening free will beliefs are considered to be caused by the disfunction of self-control (or self-regulation) processes. Neurocognitive research reported that weakening free will beliefs had undermined some fundamental processes such as inhibition (Rigoni et al., 2012) or monitoring (Rigoni et al., 2013, 2015).

A second approach is a correlational approach in which researchers estimate relationships between individual differences in free will beliefs and certain psychological concepts. This approach has revealed that individual differences in free will beliefs are positively correlated with job success and life satisfaction (Stillman et al., 2010), trait self-control and academic performance (Feldman et al., 2016), and self-esteem (Rakos et al., 2008; Spronken et al., 2019). Free will beliefs enable people to trust that they have a capacity to execute their chosen behaviors, which in turn, promotes positive aspects of self-processes (i.e., self-control and self-evaluation). Results of psychophysiological research on reversal learning tasks supported that having a free will belief is beneficial for self-regulation about strategic transition (Goto et al., 2018).

As discussed in philosophy and law, psychological and neurocognitive research has revealed that free will beliefs can be related to the attitude toward criminal punishment. Experimental research reported that weakening free will beliefs reduced retributive punishment (Shariff et al., 2014). Correlational research also revealed that individual differences in free will beliefs were positively correlated with intolerance to unethical behaviors and agreement with severe criminal punishment (Krueger et al., 2014; Martin et al., 2017). Free will beliefs seem to encourage the notion that people should be responsible for their behaviors (Nahmias et al., 2005), which can be supported by the results that free will beliefs are positively correlated with correspondence bias (Genschow et al., 2017; Genschow and Vehlow, 2019) and conservative attitude, such as the belief in a just world (Carey and Paulhus, 2013).

### Japanese Translations of the FAD-Plus

The FAD-Plus was developed from the standpoint of compatibilism; thus, it can assess belief in free will along with a deterministic worldview independently. The FAD-Plus consists of 27 items in a five-point Likert format. The subscales of the FAD-Plus are free will (seven items), scientific determinism (seven items), fatalistic determinism (five items), and unpredictability (eight items). The subscale “free will” directly assesses free will beliefs, the degree to which individuals believe in free will. The subscales “scientific determinism” and “fatalistic determinism” assess deterministic worldviews, the degree to which individuals believe that all events are completely determined by previously existing causes. These two subscales are distinguished by determinants; the former refers to scientific causality, whereas the latter refers to fatalistic inevitability. The subscale “unpredictability” assesses non-deterministic worldviews, the degree to which individuals believe that all events are not completely determined by previously existing causes. The FAD-Plus has been widely used by the free will belief researchers, and also translated into French (Caspar et al., 2017), Polish (Kondratowicz-Nowak et al., 2018), and Chinese (Liu et al., 2020).

There also exist two Japanese-translated versions of FAD-Plus independently developed by other researchers: FAD+ (Watanabe et al., 2014) and FAD-J (Goto et al., 2015). Both FAD+ and FAD-J were back-translated, and their factorial and construct validities were tested through large-scale surveys (for FAD+, the surveys were conducted on university students (*N* = 203) in Study 1 and registered monitors of a web survey company (*N* = 362) in Study 2; for FAD-J, both surveys were conducted on registered monitors of a web survey company (*N* = 3,000) in Study 1 and (*N* = 416) in Study 2). Both researchers confirmed that each scale consists of four subscales like the original scale, FAD-Plus (i.e., free will, scientific determinism, fatalistic determinism, and unpredictability). They also reported that reliability coefficients for all subscales were acceptable (Cronbach's αs ≥0.64). The construct validity (i.e., convergent validity and discriminant validity) of both scales was confirmed mainly by their correlations with the locus of control. In Goto et al. (2015), only free will was positively correlated with the internal locus of control, and the other three scales were positively correlated with the external locus of control. Moreover, only fatalistic determinism was negatively correlated with the internal locus of control. These results replicated the correlation analysis reported in Paulhus and Carey (2011). Although Watanabe et al. (2014) used a unidimensional scale of locus of control, the results are consistent with those in Paulhus and Carey (2011). In Watanabe et al. (2014), free will was positively correlated with the (internal-external) locus of control, and scientific determinism and fatalistic determinism were negatively correlated with it.

These translations have helped Japanese researchers examined the psychological functions of free will beliefs. In terms of its relation to self-control, researchers have revealed that free will beliefs are associated with the monitoring process on decision-making (Goto et al., 2018) and the valuing process on personal goals and desires (Ozaki et al., 2017). In terms of its relation to criminal punishment, researchers have revealed that free will beliefs are associated with moral responsibility (Kasahara et al., 2017; Matsuki et al., 2021).

However, no research has directly compared whether FAD+ and FAD-J can assess the same construct. While it is sometimes argued that different scales can assess the same construct, it is less often directly discussed whether different translated scales can assess the same construct. Since the wordings of the two translations are different, the possibility of translation-related artifacts in the scoring processes cannot be ruled out. As an example of how translation can change the factorial structure of the FAD-Plus, Kondratowicz-Nowak et al. (2018) assumed the Polish-translated FAD-plus as a scale consisting of three factors. Although both of the two Japanese-translated scales have been validated to assess the four factors, Watanabe et al. (2014) removed some items during the validation process of the FAD+ and reported the full-item version as reference information without detailed results “for use in international comparisons.” Thus, a direct comparison of the psychometric properties of these two translations is necessary to consider whether the scores assessed by them can be interpreted interchangeably.

### Research Questions

The aim of the present study is to investigate whether two Japanese translations of FAD-Plus (i.e., FAD+ and FAD-J) have similar psychometric features. Based on the validity aspect frequently focused on when constructing psychological scales, the following three research questions were formulated. The first question is whether both two scales consist of the same four subscales. When creating a translation of a foreign scale, researchers test whether the same factor structure of the original scale is reproduced. Therefore, it is important to address the first question to ensure that the scale of the translated version adequately reflects the content of the original scale.

The second question is whether respondents respond similarly to the items translated from the same original items. When creating a translated version of a scale, back-translation is done to ensure that the translated items retain the meaning of the items in the original scale. In the comparison of the Japanese translated scale, we thought it was also important to check whether each pair of items (i.e., two different items translated from the same original item) expressed the same meaning. In the present study, we approach this question by focusing on the similarity of responses for each pair of items.

The third question is whether both scales can assess the functionally same construct. As with the factor structure, it is an important issue to consider when examining validity as to whether there can be observed correlations between the assessed scores and other indicators similar to those reported in previous research. Therefore, in this study, we also test the construct validity of these two translated scales by comparing the correlations of free will and determinism beliefs and other criteria between them. As the external criteria, we focus on the locus of control, which is often used as the anchor of the construct validity of free will beliefs (e.g., Paulhus and Carey, 2011; Liu et al., 2020). Locus of control refers to the belief that individuals attribute the controllability of their behaviors to internal or external (i.e., others or chances) forces (Rotter, 1966; Levenson, 1973). Paulhus and Carey (2011) have claimed that free will and determinism beliefs might be confused with, but not the same as internal and external locus of control, respectively. As free will beliefs include internal locus of control, these two concepts are expected to be positively correlated. Although both scientific determinism and fatalistic determinism are expected to be positively correlated with an external locus of control, only fatalistic determinism is expected to be negatively correlated with an internal locus of control. The basis of this expectation is that scientific determinism subsumes both internal forces (e.g., biological makeup, genes) and external forces (e.g., environments, the laws of nature), although fatalistic determinism attributes controllability to external forces (e.g., fate, destiny). Unpredictability tends to be positively correlated with an external locus of control in previous research (Paulhus and Carey, 2011; Liu et al., 2020). Thus, we should examine the pattern of correlation among subscales of FAD-Plus and locus of control to test the convergent validity and the discriminant validity between similar concepts (i.e., free will and unpredictability/scientific determinism and fatalistic determinism).

In addition to the locus of control, we focused on three other concepts as the external criteria of construct validity: trait self-control, self-esteem, and belief in a just world. Previous research has revealed that free will beliefs were positively correlated with self-control (self-regulation), positive self-evaluations (e.g., self-esteem, life satisfaction), and the attitude toward criminal punishment, as reviewed in “The Psychological Function of Free Will Beliefs” section. To test the convergent validity of free will beliefs, it is important to test whether the survey using the translated scales can replicate these findings. We expected that free will beliefs would be positively correlated with trait self-control, individual differences in the capability of self-control accomplishment (Tangney et al., 2004), and self-esteem. We also expected that free will beliefs would be positively correlated with belief in a just world, which makes individuals believe that good people are rewarded and bad people are punished as the world is both just and fair (Lerner, 1971). Although scientific determinism and fatalistic determinism may be negatively correlated with these three concepts, we did not make any predictions in advance as free will and determinism are not opposite concepts under compatibilism, the premise of the FAD-plus.

## Materials and Methods

### Participants

Data were collected on CrowdWorks (https://crowdworks.jp/), an Internet platform of a crowdsourcing service that allows people (mainly Japanese) to complete online tasks. We planned to collect responses of 800 people older than 18 years and fluent in reading Japanese. As described in the later section, we collected data from two different samples. Following the rule of thumb for factor analysis (10 people for each item), we considered that we should obtain the data from 270 participants, at a minimum, for each sample. As previous studies (e.g., Miura and Kobayashi, 2018) reported that almost 15% of Japanese respondents failed to respond to the directed questions scale (DQS) (Maniaci and Rogge, 2014), we needed to collect 320 [estimated by 270/ (100-15) ×100] responses for each sample. Considering the possibilities of unexpected errors in data collection, we aimed to collect up to 400 responses for each sample. Although we decided to stop collecting the data after obtaining over 640 responses in a week, we were able to collect a total of over 800 responses within a week.

Finally, we collected the data from 839 participants (421 for Sample A and 416 for Sample B). As 37 participants (4.4%) failed to respond to the DQS, we analyzed the data from 802 participants (403 for Sample A and 399 for Sample B; 268 males, 540 females, and 10 unspecified; mean age = 38.15, SD = 10.62, range = 18–80 years).

### Procedure

After participants provided information about their age and gender, they were asked to complete six scales: FAD-J, FAD+, the locus of control scale, the Rosenberg self-esteem scale, the brief self-control scale, and the global belief in a just world scale. The order of FAD-J and FAD+ was counter-balanced in Sample A and Sample B. The participants in sample A completed FAD-J first and then completed FAD+. In contrast, the participants in sample B completed FAD+ first and then completed FAD-J.

### Japanese-Translated Scales of the FAD-Plus

FAD-J (Goto et al., 2015) and FAD+ (Watanabe et al., 2014) are the Japanese translations of the free will and determinism scale, originally developed by Paulhus and Carey (2011). These two scales have the same structure, although the detailed descriptions in the items were different. Each scale consists of 27 items in a five-point Likert format with anchors of 1 = まったくあてはまらない (“*Strongly disagree*”) to 5 = とてもよくあてはまる (“*Strongly agree*”). The four subscales are free will (seven items), scientific determinism (seven items), fatalistic determinism (five items), and unpredictability (eight items). Watanabe et al. (2014) developed FAD+ with 17 items by omitting items with small loading values through factor analysis. However, as the following research (Watanabe et al., 2016) has used FAD+ as the 27-item scale, we also used FAD+ as the 27-item scale. All items in the original scale (FAD-Plus) and translated scales (FAD+ and FAD-J) are listed in the Supplementary Table 1.

### External Criteria for Testing Construct Validity

The locus of control scale (Hazama et al., 2000) is the Japanese translation of the scale developed by Shewchuk et al. (1992). Hazama et al. (2000, 2001) have confirmed that this scale has the same factor structure as the original scale by conducting multiple surveys on different samples. Although Hazama et al. (2001) reported internal consistency of each subscale was relatively low (Cronbach's alpha ≥0.52), the following research has reported sufficient values (≥0.71) from a wider range of samples (Goto et al., 2015). The following research has also shown that the scores of this scale were correlated with self-efficacy (Tabara et al., 2000); thus, it can be interpreted as a scale that appropriately assesses the locus of control. This scale consists of seven items in a seven-point Likert format with anchors of 1 = 全く違 うと思う (“*Strongly disagree*”) to 7 = 全くそう思う (“*Strongly agree*”). There are two subscales, external (four items) and internal (three items).

The Rosenberg self-esteem scale (Rosenberg, 1965) is widely used to assess trait self-esteem. In this study, we used the Japanese-translated scale developed by Mimura and Griffiths (2007). This scale was back-translated, and the factorial structure and reliability (Cronbach's alpha = 0.81) were tested through a large-scale survey on Japanese-speaking and English-speaking populations. The following research has tested the criterion-related validity of this scale from the viewpoint of correlation with self-scheme, depression (automatic thoughts), and happiness (Uchida and Ueno, 2010). This scale consisted of 10 items in a four-point Likert format with anchors of 1 = 強 くそう思わない (“*Strongly disagree*”) to 4 = 強 くそう思う (“*Strongly agree*”).

The brief self-control scale (Ozaki et al., 2016) is the Japanese translation of the scale developed by Tangney et al. (2004). This scale was back-translated, and the factorial structure and reliability (Cronbach's alpha ≥0.75) were tested through multiple surveys on wide-range samples. The criterion-related validity was also tested from the viewpoint of correlation with other scales about self-regulation and a cognitive task (a stop-signal task). The brief self-control scale is widely used to assess trait self-control. This scale consists of 13 items in a five-point Likert format with anchors of 1 = 全くあてはまらない (“*Not at all*”) to 5 = とてもあてはまる (“*Very much*”).

The global belief in a just world scale (Shirai, 2010) is the Japanese translation of the scale developed by Lipkus (1991). Shirai (2010) has reported this translated scale was sufficiently reliable (Cronbach's alpha = 0.73) and correlated with the locus of control as the previous research has reported. This scale consists of seven items in a six-point Likert format with anchors of 1 = 全くそう思わない (“*Strongly disagree*”) to 6 = 非常にそう思う (“*Strongly agree*”).

### Analytical Plan

Based on our three research questions, we analyzed our data as below. To ensure fair comparisons of the two translated scales, research procedures and analytical plans were preregistered on https://osf.io/798sh/. Instances in which the plan has been changed from the preregistered plan are noted. The datasets for this manuscript are also available on https://osf.io/2xbe5/.

#### Comparing the Factor Structures

First, we conducted confirmatory factor analysis (CFA) to evaluate the factor structure of the FAD-J in Sample A and that of the FAD+ in Sample B separately. As an additional analysis to the preregistered plan, we also conducted CFA to evaluate both FAD-J and FAD+ using the entire dataset to avoid sample-dependent bias. To evaluate overall model adequacy, we used CFI (≥0.90), SRMR (≤ 0.08), and RMSEA (≤ 0.08) following Brown (2006) or ≥0.80 for CFI as loose criteria, following the fit indices obtained in Paulhus and Carey (2011).

Second, if the results showed that the original model of FAD-Plus demonstrates an adequate fit in each scale, we conducted multigroup CFA to test the measurement and structural invariance across the FAD-J in Sample A and the FAD+ in Sample B. For the first step, we examined configural invariance (whether the same number of factors best represents each scale). Next, we examined metric invariance (whether factor loadings are equal for both scales). Then, we investigated the scalar invariance (whether factor loadings and intercepts are equal). Finally, we assessed structural invariance (whether factor variances and covariance are equal). To evaluate these invariances, we used the changes in CFI (≤ 0.005), following Chen (2007).

#### Comparing the Item-Level Psychometric Characteristics

First, by using the whole sample, we calculated Pearson correlations between the responses on items translated from the same original item. If the meanings of items in both scales are identical, correlation coefficients will be extremely high (≥0.90).

Then, we fit the generalized partial credit model of item response theory (Muraki, 1992) for each subscale by using the whole sample. If the participants responded to each scale similarly, the estimated parameters would be similar between the items translated from the same original item.

#### Comparing the Functionalities

We compared the correlation coefficients among belief in free will and determinism, locus of control, trait self-control, self-esteem, and belief in a just world between two Japanese-translated scales. If these two scales do not assess the functionally same construct, the correlation coefficients between subscales and other constructs would significantly differ in these two Japanese-translated scales (*p* < 0.05).

## Results

### Factor Structures of FAD+ and FAD-J

First, we conducted CFA to evaluate the factor structure of the FAD-J and the FAD+. We reported the fit indices obtained by both analyzing Sample A and Sample B separately and analyzing the entire dataset in Table 1. Results showed that the SRMR and RMSEA met the preregistered criteria (≤ 0.080) in both scales, although CFI did not (≥0.95, or ≥0.80 as loose criteria). This may indicate that the theoretical model and the scale themselves should be modified. However, as some researchers have questioned using the absolute values of CFI as a fit index of a factor model, which reflects not only the model fit but also the reliability of scales (Moshagen and Auerswald, 2018), we move on to the next step of the analysis and discuss this point later.

**Table 1:** The fit indices of confirmatory factor analysis of FAD+ and FAD-J.

	**Separately**		**Whole dataset**	
	**FAD-J (Sample A:**	**FAD+ (Sample B:**	**FAD-J (Whole sample:**	**FAD+ (Whole sample:**
	***n* = 403)**	***n* = 399)**	***N* = 802)**	***N* = 802)**
χ^2^, *df, p*-value	χ^2^(318) = 861.99, *p* <0.05	χ^2^(321) = 752.76, *p < * 0.05	χ^2^(318) = 1503.48, *p < * 0.05	χ^2^(321) = 1391.75, *p < * 0.05
CFI	0.688	0.777	0.742	0.775
RMSEA	0.065	0.059	0.068	0.065
SRMR	0.078	0.079	0.079	0.080

Next, we conducted multigroup CFA to test the measurement and structural invariance across the FAD-J in Sample A and the FAD+ in Sample B. We reported fit indices obtained by multigroup CFA on the configural invariance model, the metric invariance model, the scalar invariance model, and the structural invariance model in Table 2. Results showed that the CFI of the configural invariance model is larger than those in the other three models by more than the criteria (0.005). This indicates that there exist differences in factor loadings, inter-factor covariance, and mean structures between the FAD-J and the FAD+.

**Table 2:** The fit indices of multigroup confirmatory factor analysis of FAD+ and FAD-J.

	**χ^**2**^, *df*, *p*-value**	**CFI**	**RMSEA**	**SRMR**	**AIC**	**ΔCFI (vs. Configural invariance model)**
Configural invariance	χ^2^(636) = 1614.74, *p < * 0.05	0.735	0.062	0.075	55827.20	
Metric invariance	χ^2^(659) = 1684.88, *p < * 0.05	0.723	0.062	0.077	55851.34	−0.012
Scalar invariance	χ^2^(659) = 1684.88, *p < * 0.05	0.723	0.062	0.077	55851.34	−0.012
Structural invariance	χ^2^(665) = 1698.21, *p < * 0.05	0.721	0.062	0.078	55852.66	−0.014

### Comparing Item-Level Psychometric Characteristics of FAD+ and FAD-J

First, we reported correlation coefficients between item pairs translated from the same original items in Table 3. Results showed that, although all pairs were positively intercorrelated, the correlation coefficients were not sufficiently strong to conclude that the meanings of items in both scales are the same (>0.90). Moreover, the correlation coefficients were extremely small in items 4 (free will: “*People have complete control over the decisions they make*.”) and 15 (unpredictability: “*People are unpredictable*.”). These two items seem to be translated into almost different meanings.

**Table 3:** Correlation coefficients between item pairs translated from the same items.

**Items**		***r***	**95%CI**	
			**Lower bound**	**Upper bound**
1	I believe that the future has already been determined by fate.	0.680	0.641	0.716
2	People's biological makeup determines their talents and personality.	0.427	0.368	0.482
3	Chance events seem to be the major cause of human history.	0.559	0.509	0.605
4	People have complete control over the decisions they make.	0.153	0.085	0.220
5	No matter how hard you try, you can't change your destiny.	0.533	0.482	0.581
6	Psychologists and psychiatrists will eventually figure out all human behavior.	0.769	0.739	0.795
7	No one can predict what will happen in this world.	0.594	0.548	0.637
8	People must take full responsibility for any bad choices they make.	0.587	0.540	0.631
9	Fate already has a plan for everyone.	0.670	0.629	0.706
10	Your genes determine your future.	0.592	0.545	0.635
11	Life seems unpredictable - just like throwing dice or flipping a coin.	0.570	0.521	0.615
12	People can overcome any obstacles if they truly want to.	0.757	0.725	0.785
13	Whatever will be, will be - there's not much you can do about it.	0.398	0.339	0.455
14	Science has shown how your past environment created your current intelligence and personality.	0.501	0.448	0.552
15	People are unpredictable.	0.299	0.234	0.360
16	Criminals are totally responsible for the bad things they do.	0.654	0.612	0.691
17	Whether people like it or not, mysterious forces seem to move their lives.	0.571	0.522	0.616
18	As with other animals, human behavior always follows the laws of nature.	0.590	0.543	0.633
19	Life is hard to predict because it is almost totally random.	0.420	0.361	0.475
20	Luck plays a big role in people's lives.	0.519	0.466	0.568
21	People have complete free will.	0.457	0.400	0.510
22	Parents' character will determine the character of their children.	0.671	0.631	0.707
23	People are always at fault for their bad behavior.	0.325	0.262	0.386
24	Childhood environment will determine your success as an adult.	0.609	0.563	0.650
25	What happens to people is a matter of chance.	0.366	0.305	0.425
26	Strength of mind can always overcome the body's desires.	0.581	0.533	0.625
27	People's futures cannot be predicted.	0.491	0.436	0.542

Next, we used a generalized partial credit model for each subscale separately using combined item sets (i.e., 14 items for free will, 14 items for scientific determinism, 10 items for fatalistic determinism, and 16 items for unpredictability). Estimated coefficients are reported in Table 4. There seems to be a difference in the threshold in items 4, 21 (free will), 18, 24 (scientific determinism), 13 (fatalistic determinism), 15, and 20 (unpredictability). Moreover, there appears to be a difference in discrimination in item 15 (unpredictability). Thus, almost 25% of the item pairs have different characteristics of assessment.

**Table 4:** Estimated parameters in the generalized partial credit model for each subscale.

	**Threshold**				**Discrimination**
	**1|2**	**2|3**	**3|4**	**4|5**	
**Free will**					
FAD-J item4	−5.205	−2.885	−2.351	2.501	0.494
FAD+ item4	−4.690	0.707	2.262	8.837	0.326
FAD– J item8	−2.818	−1.457	−0.656	2.103	1.009
FAD+ item8	−2.837	−1.287	−0.450	1.794	1.323
FAD– J item12	−4.626	−0.625	0.529	6.031	0.316
FAD+ item12	−3.445	−1.326	0.649	5.445	0.339
FAD– J item16	−3.806	−1.765	−1.144	0.514	1.416
FAD+ item16	−3.315	−1.471	−1.090	0.660	1.227
FAD– J item21	−6.751	−2.054	−1.406	4.186	0.501
FAD+ item21	−3.986	−1.262	−0.443	2.708	0.534
FAD– J item23	−4.391	−1.130	−0.555	2.623	0.787
FAD+ item23	−4.344	−1.398	−0.044	2.878	0.918
FAD– J item26	−3.779	−0.592	−0.432	5.481	0.455
FAD+ item26	−4.231	−0.170	0.347	5.484	0.482
**Scientific determinism**					
FAD– J item2	−2.943	−0.944	0.159	3.549	0.813
FAD+ item2	−3.769	−0.819	0.111	4.038	0.792
FAD– J item6	−3.007	1.828	4.127	7.992	0.281
FAD+ item6	−3.069	1.944	3.862	8.445	0.283
FAD– J item10	−1.646	0.127	0.933	3.397	1.222
FAD+ item10	−1.874	−0.196	0.542	3.246	1.258
FAD– J item14	−4.565	−1.537	0.175	6.371	0.461
FAD+ item14	−4.577	−1.778	0.097	5.235	0.554
FAD– J item18	−6.107	−0.530	0.788	7.457	0.325
FAD+ item18	−6.876	−0.450	0.803	5.890	0.338
FAD– J item22	−2.581	−1.339	−0.661	2.255	1.079
FAD+ item22	−2.717	−1.057	−0.410	2.633	1.099
FAD– J item24	−2.651	−0.765	−0.403	2.288	0.907
FAD+ item24	−2.991	−1.570	−1.077	1.715	1.018
**Fatalistic determinism**					
FAD– J item1	−1.171	0.125	1.037	2.477	2.381
FAD+ item1	−1.062	0.264	1.055	2.061	2.711
FAD– J item5	−2.028	0.692	1.365	3.196	0.929
FAD+ item5	−2.366	0.430	1.279	2.519	0.828
FAD– J item9	−0.929	0.307	1.071	2.244	2.692
FAD+ item9	−1.131	0.106	1.032	2.213	2.384
FAD– J item13	−3.370	0.663	0.843	4.155	0.620
FAD+ item13	−5.158	−0.936	−0.865	3.846	0.440
FAD– J item17	−4.286	−1.581	−1.179	2.783	0.589
FAD+ item17	−3.744	−1.568	−1.337	4.492	0.430
**Unpredictability**					
FAD– J item3	−5.211	−1.804	−0.268	3.686	0.480
FAD+ item3	−4.811	−2.232	−0.381	3.881	0.549
FAD– J item7	−3.514	−1.430	−1.000	0.492	1.753
FAD+ item7	−3.153	−1.572	−1.225	0.416	1.664
FAD– J item11	−3.251	−1.502	−1.064	1.028	1.610
FAD+ item11	−3.052	−1.222	−1.037	1.026	1.587
FAD– J item15	−8.007	−2.584	−0.784	4.297	0.413
FAD+ item15	−4.010	−1.719	−1.267	1.069	1.405
FAD– J item19	−4.412	−0.928	−0.429	2.343	0.823
FAD+ item19	−3.314	−1.091	−0.658	1.901	1.258
FAD– J item20	−5.575	−2.179	−0.321	4.261	0.413
FAD+ item20	−5.307	−2.920	−2.134	2.707	0.449
FAD– J item25	−3.861	−1.714	0.098	4.233	0.605
FAD+ item25	−4.456	−0.961	0.074	3.460	0.508
FAD– J item27	−3.481	−1.630	−1.134	0.807	1.306
FAD+ item27	−3.270	−1.188	−1.213	0.865	1.559

### Correlation Analysis Among Subscales of Translated FAD-Plus and External Criteria

We calculated the trait score by averaging responses of items in each (sub)scale. We reported the mean, SD, and Cronbach's alpha of all (sub)scales, as shown in Table 5. In both the FAD+ and FAD-J, the average score of free will is higher than that of scientific determinism and fatalistic determinism. These differences suggest that, on average, people believe in the existence of free will over deterministic world views. This is consistent with the trends observed when using the original FAD-Plus (Paulhus and Carey, 2011). It should be noted that the differences in the FAD+ are smaller than those in the FAD-J and the original FAD-Plus (the effect sizes of differences between free will and scientific determinism were d = 0.958 in the FAD-J and d = 0.310 in the FAD+; the effect sizes of differences between free will and fatalistic determinism were d = 1.277 in the FAD-J and d = 0.610 in the FAD+).

**Table 5:** Mean, SD, and Cronbach's alpha of all scales.

**Scales**		**Mean**	***SD***	**Cronbach's α**
FAD-J	Free will	3.498	0.507	0.627
	Scientific determinism	2.998	0.536	0.649
	Fatalistic determinism	2.724	0.691	0.761
	Unpredictability	3.567	0.549	0.756
FAD+	Free Will	3.241	0.546	0.662
	Scientific Determinism	3.076	0.517	0.642
	Fatalistic Determinism	2.865	0.680	0.711
	Unpredictability	3.695	0.576	0.794
Locus of control	External	4.027	1.016	0.720
	Internal	4.849	0.880	0.643
Self-esteem		2.377	0.577	0.902
Trait self-control		3.024	0.670	0.848
Beliefs in a just world		3.220	0.706	0.773

*N = 802*.

Next, we reported inter-subscale correlation coefficients of the FAD-J and FAD+, as shown in Table 6. Inter-subscale correlation coefficients were almost positive in the FAD+, although some were smaller than 0.10 in the FAD-J. By using a statistical test, significant differences in the correlation coefficient of free will and fatalistic determinism between FAD-J and FAD+ (Z = 2.865, *p* < 0.05) were found. These two subscales were positively (although weakly) correlated in the FAD+, but not significant in the FAD-J.

**Table 6:** Inter-subscale correlation coefficients of the FAD-J and the FAD+.

	**Free**	**Scientific**	**Fatalistic**	**Unpredictability**
	**will**	**determinism**	**determinism**	
Free will		0.133*	0.125*	0.103*
Scientific determinism	0.119*		0.446*	0.228*
Fatalistic determinism	**−0.018**	0.484*		0.338*
Unpredictability	0.086*	0.152*	0.306*	

*p < 0.05. Scores over the diagonal line indicate inter-subscale correlation coefficients of FAD+ (N = 802). Scores under the diagonal line indicate inter-subscale correlation coefficients of FAD-J (N = 802). The difference in correlation coefficients between free will and fatalistic determinism (in bold) is significant between the FAD+ and the FAD-J.

We also calculated correlation coefficients between the subscales of the FAD-J and the FAD+ and other scales, as shown in Table 7. Most of the correlation coefficients were similar between the FAD-J and the FAD+. Both scales generally replicated the results obtained in previous research. Consistent with Paulhus and Carey (2011), the internal locus of control was positively correlated with free will, but negatively correlated with fatalistic determinism. Accordingly, the external locus of control was positively correlated with scientific determinism, fatalistic determinism, and unpredictability. Free will was also positively correlated with self-esteem (Rakos et al., 2008; Spronken et al., 2019), trait self-control (Feldman et al., 2016), and the belief in a just world (Carey and Paulhus, 2013). Interestingly, scientific determinism was positively correlated with the belief in a just world, although fatalistic determinism was not significantly correlated with it. Although it should be noted that it was an unpredicted result, given that the belief in a just world is positively correlated with the internal locus of control (Lipkus, 1991), it can be interpreted as a result that supports the discriminant validity that only scientific determinism might attribute controllability to internal forces, unlike fatalistic determinism.

**Table 7:** Correlation coefficients of subscales of the FAD-Plus and other scales.

	**Locus of control**		**Self-esteem**	**Trait self-control**	**Belief in a just world**
	**External**	**Internal**			
Free will	−0.008/0.006	0.411*/0.368*	0.125*/0.074*	0.161*/0.150*	0.335*/0.361*
Scientific determinism	0.384*/0.375*	*−0.003/0.030*	−0.098*/−0.093*	−0.064/ −0.076*	0.148*/0.131*
Fatalistic determinism	0.373*/0.328*	−0.150*/−0.060	−0.213*/−0.176*	−0.044/−0.060	0.020/0.093*

*p < 0.05. N = 802. The value on the left is the correlation with the subscales of the FAD-J, and the value on the right is the correlation with the subscales of FAD+. Italicized cells indicate that the difference in correlation coefficients is marginally significant.

When focusing on the difference in correlation coefficients between two scales, free will was positively correlated with self-esteem in the FAD-J, but weekly correlated in the FAD+ (although the difference is not significant, Z = 1.021, *p* = 0.33). Moreover, fatalistic determinism is negatively correlated with the internal locus of control in the FAD-J, but not significant in the FAD+ (the difference is so-called marginally significant, Z = 1.831, *p* = 0.07). These results indicate that the FAD-J and the FAD+ can assess almost similar beliefs in terms of functionality, although some differences may exist.

## Discussion

This study investigated whether two Japanese translations of the FAD-Plus have similar psychometric properties. The results of confirmatory factor analysis support that both the FAD+ and the FAD-J have the same four-factor structure as the original FAD-Plus. The low values of CFI may be due to the unspecified covariance between some items. Paulhus and Carey (2011), who developed the original scale of FAD-Plus, assumed a covariance relationship between some of the items to increase the fit index. Caspar et al. (2017) utilized similar procedures when estimating the fit indices of factor structure in a translated version of the FAD-Plus. Such a covariance relationship can be also linked to the low [but almost equal to those reported in Goto et al. (2015) and Watanabe et al. (2014)] value in the reliability indices (i.e., Cronbach's alpha). Considering the bandwidth-fidelity dilemmas, local correlations found in scale items are an issue that should be addressed to establish a well-validated scale of free will beliefs. However, given the aim of this study, we believe that it should not significantly affect the conclusion.

There exist differences in the characteristics of assessment of each item between the FAD+ and the FAD-J. Results of multigroup CFA suggested that estimated values in factor analysis in these two scales are not equal. Since different researchers translated the FAD-Plus independently, it is possible that the meaning of the translated items may differ. Results of correlation analysis of item pairs suggested that such differences in meaning are particularly large in two item pairs. In addition, the results of the generalized partial credit model suggested that almost 25% of item pairs have different characteristics of assessment. These two analyses are common in that there exists a large difference in the translation of item 15 (unpredictability: “*People are unpredictable*”).

While the scores assessed by both scales generally showed the same trend, there were also minor differences. The difference in means of subscales showed that free will had a higher mean value than determinisms for both scales, which was consistent with the results of the original FAD-Plus (Paulhus and Carey, 2011). However, mean differences tended to be smaller in the FAD+ than the FAD-J. The results of correlation analysis among the subscales also showed that the FAD+ differed from the FAD-J and the original FAD-Plus in that there was a positive correlation between free will and fatalistic determinism. For FAD+, it may be somewhat difficult to distinguish the difference between free will and determinism when compared to the FAD-J and the original FAD-Plus.

These differences in discriminability may cause some contamination in examining the psychological functions of these beliefs. Correlation analysis with the locus of control supported those subscales of both the FAD-J and the FAD+ had sufficient convergent validity. When focusing on the other three concepts (i.e., trait self-control, self-esteem, and the belief in a just world), results also conceptually replicated the previous findings of positive relationships between free will beliefs and self-control, self-esteem, and attitude toward criminal punishment. However, as for the discriminant validity, it seems that the FAD-J is better able to distinguish scientific determinism and fatalistic determinism than the FAD+. Fatalistic determinism in FAD-J was negatively correlated with an external locus of control, but not in FAD+. Although the difference between the FAD+ and the FAD-J in terms of correlation with the external criteria was small, we should continue to pay attention to this possibility.

The results of the present study contribute to future research using the Japanese-translated version of the FAD-Plus in three ways. First, the differences between the FAD+ and the FAD-J are not likely to be large enough to affect conclusions as long as full items are used and correlations with other variables are examined. The FAD-J tends to be slightly more likely to replicate the correlations obtained in previous studies. However, when focusing on the mean difference in scores, it should be noted that the mean difference tends to be smaller in the FAD+. Second, the use of a selected item from the FAD+ and the FAD-J does not guarantee that the assessed scores will be equal to those assessed when full items are used. As psychometric characteristics are not similar in some item pairs, translation-induced bias may be present. Third, as a secondary contribution, the results of the present study can also be used to link the FAD+ and the FAD-J. As there are two different translations of the same original scale, these scales could be used as a parallel test. It would be helpful for future research if we could utilize the fact that there are two different translations rather than consider which is the better scale with a competitive perspective.

The establishment of valid assessment tools can contribute not only to the promotion of correlational research but to the establishment of valid experimental manipulations. Some of the past findings of free will beliefs have been recently identified as being subject to reproducibility problems. Some researchers attempted to directly replicate the effect of weakening free will beliefs but reported that this manipulation caused no effects on moral behavior or punishment judgments (Monroe et al., 2017; Nadelhoffer et al., 2020). Meta analytical results also showed that weakening free will beliefs has little effect on behaviors or judgments beyond the belief itself (Genschow et al., 2021). Thus, the findings of the experimental approach have been somewhat questioned. In contrast, the findings of the correlational approach are inferred to be relatively robust, as some results were obtained from analyzing large-scale surveys (e.g., Martin et al., 2017). Crone and Levy (2019) pointed out that there may be some moderator effects due to some situational or personal factors. In any case, including Japanese translation ones, well-validated and internationally comparable measurement to assess free will belief is necessary for the accumulation of evidence in this research area.

The validity of psychological scales should be continuously tested; thus, it is not appropriate to consider the comparison of these two scales as complete. First, the present study did not conduct a direct comparison with the original FAD-Plus. It would be necessary to examine which translations have closer psychometric properties to the original scale. Second, the present research assumes that original FAD-Plus is a valid scale to assess free will and determinism scales, and a merely translated version can be suitable to assess the same beliefs in the Japanese population. However, a more appropriate scale that relies on Japanese culture to assess free will and determinism beliefs could be created. Although translated versions may be easier to use for international comparisons, the question of how to measure beliefs in a valid way should always be considered.

In non-English speaking countries, such as Japan, it is sometimes the case that there are multiple translations of the same original scale developed in English-speaking countries. Although multiple translations are often produced independently, it is rarely discussed whether these scales have equivalent psychometric properties. Comparing the psychometric properties of different translations may be useful for integrating research findings using these scales and for conducting research that takes advantage of the different translations (e.g., using them as parallel tests). This study may also serve as a model case for psychological measures other than free will beliefs.

## Data Availability Statement

The datasets presented in this study can be found in online repositories. The names of the repository/repositories and accession number(s) can be found at: https://osf.io/2xbe5.

## Ethics Statement

The studies involving human participants were reviewed and approved by Institutional Review Board, The University of Shiga Prefecture. The patients/participants provided their written informed consent to participate in this study.

## Author Contributions

TG contributed to the conception and design of the study, conducted the study (i.e., data collection and analysis), and then wrote the manuscript.

## Funding

This research was supported by JSPS KAKENHI 19K14361.

## Conflict of Interest

The author declares that the research was conducted in the absence of any commercial or financial relationships that could be construed as a potential conflict of interest.

## Publisher's Note

All claims expressed in this article are solely those of the authors and do not necessarily represent those of their affiliated organizations, or those of the publisher, the editors and the reviewers. Any product that may be evaluated in this article, or claim that may be made by its manufacturer, is not guaranteed or endorsed by the publisher.
