# Supplementary material for: Comparing the Psychometric Properties of Two Japanese-Translated Scales of the Free Will and Determinism-Plus Scale
Source: Front Psychol. 2021 Oct 5;12:720601. doi: 10.3389/fpsyg.2021.720601 (PMC8523834; doi:10.3389/fpsyg.2021.720601)
Supplement: Supplementary file 1 [file Table_1.docx]

**SUPPLEMENTARY INFORMATION**

**Comparing the Psychometric Properties of Two Japanese-Translated Scales of the Free Will and Determinism-Plus Scale**

Takayuki Goto

<https://doi.org/10.3389/fpsyg.2021.720601>

Table S1. All items in the original scale (the FAD-Plus) and translated scales (the FAD+ and the FAD-J).

| items | | scale |
| --- | --- | --- |
| 1 | I believe that the future has already been determined by fate. | FAD-Plus |
|  | 未来はいつも運命によって決められていると信じている | FAD-J |
|  | 未来は運命によってすでに決定されていると思う | FAD+ |
| 2 | People's biological makeup determines their talents and personality. | FAD-Plus |
|  | 人の性格や才能は、（脳のつくりなどの）生物学的な構造によって決まっている | FAD-J |
|  | 人の生物学的な形質が、その人の才能や性格を決定する | FAD+ |
| 3 | Chance events seem to be the major cause of human history. | FAD-Plus |
|  | 人の歴史の大部分は偶然の出来事の積み重ねである | FAD-J |
|  | 偶発的な出来事が人類の歴史の主要な要因となっているように思う | FAD+ |
| 4 | People have complete control over the decisions they make. | FAD-Plus |
|  | 人は自分の意志で決定を下すことができる | FAD-J |
|  | 人々は自分自身が下す決定を完全にコントロールしている | FAD+ |
| 5 | No matter how hard you try, you can't change your destiny. | FAD-Plus |
|  | どんなに努力しても、自分の運命は変えられない | FAD-J |
|  | どんなに努力しても、みずからの宿命を変えることはできない | FAD+ |
| 6 | Psychologists and psychiatrists will eventually figure out all human behvior. | FAD-Plus |
|  | 心理学者や精神科医はやがて人のふるまいの全てを解明するだろう | FAD-J |
|  | 心理学者や精神科医は、いずれすべての人間行動を解明するだろう | FAD+ |
| 7 | No one can predict what will happen in this world. | FAD-Plus |
|  | 誰もこれから起こることを予測できない | FAD-J |
|  | この世界で何が生じるかは、誰も予測ができない | FAD+ |
| 8 | People must take full responsibility for any bad choices they make. | FAD-Plus |
|  | 人は自分が下した誤った選択に対しては、一切の責任を負わなくてはならない | FAD-J |
|  | 人々は自分がした誤った選択に対して、すべての責任を取らなければならない | FAD+ |
| 9 | Fate already has a plan for everyone. | FAD-Plus |
|  | 全ての人の人生は、最初から運命によって決められている | FAD-J |
|  | すべての人には、運命によって未来図が用意されている | FAD+ |
| 10 | Your genes determine your future. | FAD-Plus |
|  | 自分の将来は、遺伝子によって決められている | FAD-J |
|  | 遺伝子が人の将来を決定する | FAD+ |
| 11 | Life seems unpredictable - just like throwing dice or flipping a coin. | FAD-Plus |
|  | サイコロの目やコイントスのように、人生は予測できない | FAD-J |
|  | サイコロを投げたりコインをはじくように、人生は予測できないと思われる | FAD+ |
| 12 | People can overcome any obstacles if they truly want to. | FAD-Plus |
|  | 人は心から望めば、どんな障害でも乗り越えられる | FAD-J |
|  | 人々は真に望めば、どんな障害でも乗り越えることができる | FAD+ |
| 13 | Whatever will be, will be - there's not much you can do about it. | FAD-Plus |
|  | 物事はなるようにしかならず、自分にできることは少ない | FAD-J |
|  | なるようになる。それは自分にはどうしようもない | FAD+ |
| 14 | Science has shown how your past environment created your current intelligence and personality. | FAD-Plus |
|  | 過去の経験がどのように現在の自分の知性や性格を形作ってきたかを、科学は示してくれる | FAD-J |
|  | 知能や性格がその人の過去の環境によってどのように形成されるかを科学は示してきた | FAD+ |
| 15 | People are unpredictable. | FAD-Plus |
|  | 人は誰しも予測できないようなふるまいをする | FAD-J |
|  | 人は予測不可能である | FAD+ |
| 16 | Criminals are totally responsible for the bad things they do. | FAD-Plus |
|  | 犯罪者には自分の行った悪事に対する、全面的な責任がある | FAD-J |
|  | 犯罪者は自分がした悪いおこないに対してすべての責任を負う | FAD+ |
| 17 | Whether people like it or not, mysterious forces seem to move their lives. | FAD-Plus |
|  | その考え方が好きかどうかは別として、人生は説明できない力に動かされているように思う | FAD-J |
|  | 好むかどうかにかかわらず、不思議な力が人生を動かすように思う | FAD+ |
| 18 | As with other animals, human behavior always follows the laws of nature. | FAD-Plus |
|  | 人間の行動は他の動物たちと同じように、いつも自然の摂理に従っている | FAD-J |
|  | ほかの動物と同様に、人間の行動は自然の法則に常に従う | FAD+ |
| 19 | Life is hard to predict because it is almost totally random. | FAD-Plus |
|  | 日々の出来事は全くもって一貫性を持たないため、先を予測することは難しい | FAD-J |
|  | 人生はほぼすべてがランダムなため、予測することが難しい | FAD+ |
| 20 | Luck plays a big role in people's lives. | FAD-Plus |
|  | 人生は運に負うところが大きい | FAD-J |
|  | 運は人々の人生において、大きな役割を果たしている | FAD+ |
| 21 | People have complete free will. | FAD-Plus |
|  | 人は自らの自由な意志や思考で行動している | FAD-J |
|  | 人々は完全な自由意志を持っている | FAD+ |
| 22 | Parents' character will determine the character of their children. | FAD-Plus |
|  | 親の持つ性質は、子どもの性質を決めている | FAD-J |
|  | 親の特性が子どもの特性を決めるだろう | FAD+ |
| 23 | People are always at fault for their bad behavior. | FAD-Plus |
|  | 人は、自分のあやまちにいつも責任を負っている | FAD-J |
|  | 人々は自分の悪い行動について常に非がある | FAD+ |
| 24 | Childhood environment will determine your success as an adult. | FAD-Plus |
|  | 大人になってから成功するかは子どもの頃の環境で決まる | FAD-J |
|  | 幼少期の環境が、大人になったときの成功を左右する | FAD+ |
| 25 | What happens to people is a matter of chance. | FAD-Plus |
|  | 人に起こる出来事は、偶然の産物である | FAD-J |
|  | 人々に何が起こるかは運次第である | FAD+ |
| 26 | Strength of mind can always overcome the body's desires. | FAD-Plus |
|  | 精神力が強ければ、自分に生じた欲望をいつも抑えることができる | FAD-J |
|  | 意志の強さは身体的欲求にいつでも打ち勝つことができる | FAD+ |
| 27 | People's futures cannot be predicted. | FAD-Plus |
|  | 人の将来は予測することができない | FAD-J |
|  | 人々の未来は予測することができない | FAD+ |
